# Phytochemical Composition and In Vitro Antioxidant, Anti-Inflammatory, Anticancer, and Enzyme-Inhibitory Activities of *Artemisia nilagirica* (C.B. Clarke) Pamp

**DOI:** 10.3390/molecules27207119

**Published:** 2022-10-21

**Authors:** Jawaher J. Albaqami, Tancia P. Benny, Hamida Hamdi, Ammar B. Altemimi, Aswathi Moothakoottil Kuttithodi, Joice Tom Job, Anju Sasidharan, Arunaksharan Narayanankutty

**Affiliations:** 1 Department of Biology, College of Science, Taif University, P.O. Box 11099, Taif 21944, Saudi Arabia; 2 Division of Cell and Molecular Biology, PG and Research Department of Zoology, St. Joseph’s College (Autonomous), Devagiri, Calicut 673 008, Kerala, India; 3 Zoology Department, Faculty of Science, Cairo University, Giza 12613, Egypt; 4 Department of Food Science, College of Agriculture, University of Basrah, Basrah 61004, Iraq; 5 College of Medicine, University of Warith Al-Anbiyaa, Karbala 56001, Iraq

**Keywords:** phytochemistry, *Artemisia nilagirica*, *Asteraceae*, antioxidant, anti-inflammatory activity, anticancer activity

## Abstract

Plants have been employed in therapeutic applications against various infectious and chronic diseases from ancient times. Various traditional medicines and folk systems have utilized numerous plants and plant products, which act as sources of drug candidates for modern medicine. Artemisia is a genus of the *Asteraceae* family with more than 500 species; however, many of these species are less explored for their biological efficacy, and several others are lacking scientific explanations for their uses. *Artemisia nilagirica* is a plant that is widely found in the Western Ghats, Kerala, India and is a prominent member of the genus. In the current study, the phytochemical composition and the antioxidant, enzyme-inhibitory, anti-inflammatory, and anticancer activities were examined. The results indicated that the ethanol extract of *A. nilagirica* indicated in vitro DPPH scavenging (23.12 ± 1.28 µg/mL), ABTS scavenging (27.44 ± 1.88 µg/mL), H_2_O_2_ scavenging (12.92 ± 1.05 µg/mL), and FRAP (5.42 ± 0.19 µg/mL). The anti-inflammatory effect was also noticed in the Raw 264.7 macrophages, where pretreatment with the extract reduced the LPS-stimulated production of cytokines (*p* < 0.05). *A. nilagirica* was also efficient in inhibiting the activities of α-amylase (38.42 ± 2.71 µg/mL), α-glucosidase (55.31 ± 2.16 µg/mL), aldose reductase (17.42 ± 0.87 µg/mL), and sorbitol dehydrogenase (29.57 ± 1.46 µg/mL). It also induced significant inhibition of proliferation in breast (MCF7 IC_50_ = 41.79 ± 1.07, MDAMB231 IC_50_ = 55.37 ± 2.11µg/mL) and colon (49.57 ± 1.46 µg/mL) cancer cells. The results of the phytochemical screening indicated a higher level of polyphenols and flavonoids in the extract and the LCMS analysis revealed the presence of various bioactive constituents including artemisinin.

## 1. Introduction

Medicinal plants are important sources of various biologically and pharmacologically active compounds [1,2]. Several traditional medicinal plants have been shown to have strong pharmacological properties, such as radical neutralizing, inflammation-preventing, antiproliferative, hypolipidemic, hepatoprotective, neuroprotective, antithrombotic, and immunomodulatory activities [3,4]. Among the various plant families, *Asteraceae* is one of the most widely utilized ones, and it is also equipped with numerous biological and pharmacological activities. Among the various genera, the Artemesia genus is well-known [5,6,7].

The Artemisia genus and the member species are well-studied for their various biological activities [8,9,10,11,12,13]. *Artemisia annua *L. has demonstrated significant medicinal benefits because of the presence of artemisinin [14]. *Artemisia mongolica* is another important member of the genus, which is rich in lactone derivatives of Sesquiterpene and a wide range of pharmacological activities [15]. The different species of the genus were found to have strong antibacterial and antifungal properties against pathogenic organisms in humans, livestock, and plants [16,17,18,19,20]. Antiproliferative and apoptotic effects are attributed to the bioactive compounds and extracts of various species of *Artemisia* [21,22,23,24].

*Artemisia nilagirica* is distributed throughout the Western Ghats, India; it has been traditionally applied by various tribal healers in the area for the treatment of infectious diseases and toxicity prevention. The plant has been shown to have significant biological and pharmacological activities based on various in vitro and in vivo studies. The initial studies by Ahameethunisa and Hopper [25] identified the antibacterial potential of the methanol extract of *A. nilagirica* against 15 bacterial strains. Further, the extract was found to be effective against *Mycobacterium smegmatis* and *M. bovis* [26]. The extract was also found to be effective against the malarial parasite *Plasmodium falciparum* [27].

The anticancer activities of the methanol and ethyl acetate extracts were also elucidated against the human monocytic leukemia cell (THP-1) [28]. Later, studies by Sahu, Meena, Shukla, Chaturvedi, Kumar, Datta, and Arya [24] also supported these results in colorectal cancer cell models. Studies by Raju et al. [29] indicated that the anticancer activity was mediated through the inhibition of TGF-beta signaling. The plant extract was also found to inhibit inflammatory insults in human red blood cell models [30]. The fruit of *A. nilagirica* was found to have significant antiradical activity via scavenging DPPH and nitric oxide radicals [31]. The essential oil extracted from *A. nilagirica* was a rich source of monoterpenoid compounds such as thujone, and by virtue of these compounds, the essential oil inhibited the growth of various fungal pathogens [32]. The essential oil was also effective against the phytopathogenic fungal groups of table grapes [33]. Additionally, the essential oil was also effective against various bacterial populations and capable of repelling mosquitoes [34].

Although several studies have reported the preliminary pharmacological activity of the plant, there is no clear-cut information on its quantitative chemical profile and nutritional value. Additionally, the anti-inflammatory properties are yet to be discovered in cell line models, and its mechanism of action is also not specified. Therefore, the present study aimed to analyze the chemical composition of the ethanol extract of *Artemisia nilagirica* leaves in terms of the bioactive compounds and proximate composition, as well as their antioxidant potential. Further, this study for the first time attempted to analyze the enzyme-inhibitory and anti-inflammatory activities of the extract in Raw 264.7 cells stimulated by lipopolysaccharides.

## 2. Results and Discussion

### 2.1. Determination of Proximate Composition of A. nilagirica

The Artemisia species, which includes 200–400 identified plants, are extensively spread in tropical and temperate areas [6]. The importance of the artemisia species in traditional medicine is well established [5]. The plant’s antiviral, antifungal, antibiotic, insecticidal, hepatoprotective, and neuroprotective qualities make it useful in both Chinese and Ayurvedic medical systems [35]. The current study examined a specific member *Artemisia nilagirica*, its phytochemical makeup, and its pharmacological effects.

The physicochemical parameters of the *A. nilagirica* leaf powder are shown in Table 1. The predominant compounds were carbohydrate, protein, fat, and ash contents. The moisture content was estimated to be 87.4 ± 2.12%.

### 2.2. Quantitative and Qualitative Estimation of Phytochemicals in A. nilagirica

**Figure 1:**
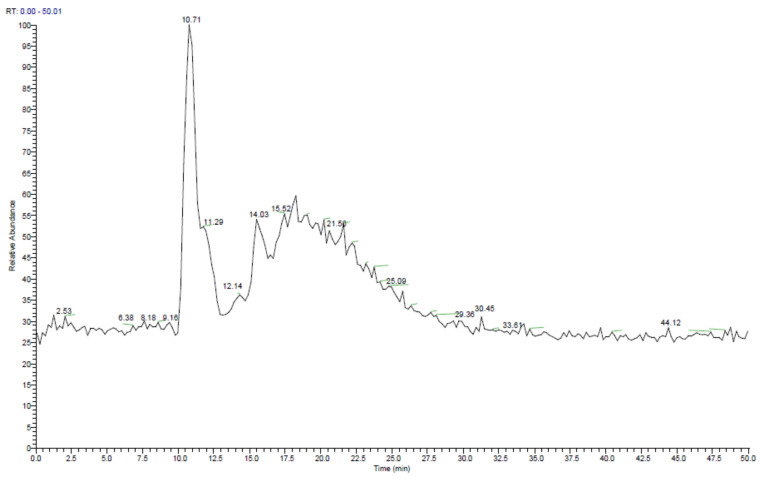
The LC-MS total ion chromatogram of the *A. nilagirica* extract.

The qualitative phytochemical screening identified the presence of compounds such as alkaloids, flavonoids, glycosides, sterols, and triterpenes (Table 2). The LCMS analysis of the *A. nilagirica* ethanol extract indicated the presence of various phytocompounds, including artemisinin, quercetin, apigenin, Β-caryophyllene, luteolin, and simple phenolic acids (Figure 1 and Table 3). Previous reports have also confirmed that numerous kinds of bioactive substances are found in *A. vulgaris*, *A. annua,* and other species, including flavonoids, sesquiterpenoids, essential oils, tannins, phenols, and saponins [15,36]. The total polyphenol content of *A. nilagirica* was estimated to be 89.51 ± 2.5 mg gallic acid equivalent/g of extract. The total flavonoid content was 14.35 ± 0.9 mg quercetin equivalent/g of extract (Table 4). Further, the HPLC quantification indicated higher levels of quercetin (240.39 ± 4.87 µg/g extract), luteolin (146.87 ± 5.29 µg/g extract), and apigenin (103.41 ± 3.35 µg/g extract) in the *A. nilagirica* extract (Table 5). These compounds are known to possess strong anti-inflammatory, antiproliferative and antidiabetic activities [37,38,39,40].

### 2.3. In Vitro Antioxidant Activities of A. nilagirica Extract

The Artemisia genus members frequently display antioxidant activity [41]; our study also confirmed the antioxidant activity of *A. nilagirica* for the first time in terms of the radical generation inhibition and reducing potentials. The IC_50_ value of the *A. nilagirica* extract in the anti-DPPH radical assay was estimated to be 23.12 ± 1.28 µg/mL. Likewise, Table 6 shows the other antioxidant activities in terms of the ABTS radical scavenging activity, hydrogen peroxide scavenging potential, and ferric-reducing antioxidant power; the respective IC_50_ values were found to be 27.44 ± 1.88, 12.92 ± 1.05, and 5.42 ± 0.19 µg/mL. On the contrary, the level of inhibition of nitric oxide radical generation (IC_50_) was determined to be 367.09 ± 12.05 µg/mL for the extract. However, in comparison with the standard antioxidant ascorbic acid (Table 6), the activity was much lower in the *A. nilagirica* extract; further purification of the extract may yield more active antioxidant compounds. The antioxidant properties are attributed to the bioactive compounds identified in the plant via LC-MS. Oxidative stress is the central independent factor that drives many chronic diseases, including cancers [42,43]; hence, the antioxidant properties of the plant may be useful in the management of diseases associated with oxidative stress.

### 2.4. Enzyme-Inhibitory Activities of A. nilagirica Ethanol Extract

The enzyme-inhibitory properties of the extract were analyzed against four enzymes involved in type 2 diabetes mellitus, including α-amylase, α-glucosidase, aldose reductase, and sorbitol dehydrogenase (Table 7). The IC_50_ values for these enzymes were 38.42 ± 2.71, 55.31 ± 2.16, 17.42 ± 0.87, and 29.57 ± 1.46 µg/mL, respectively. Furthermore, α-amylase and α-glucosidase are enzymes involved in carbohydrate metabolism and are common targets of antidiabetic drugs [44]. Similarly, the polyol pathway enzymes, including aldose reductase and sorbitol dehydrogenase, are involved in diabetic complications [45,46]. Hence, the inhibition of these enzymes could result in strong antidiabetic activity for the *A. nilagirica* extract.

### 2.5. Antiproliferative Activity of the A. nilagirica

**Figure 2:**
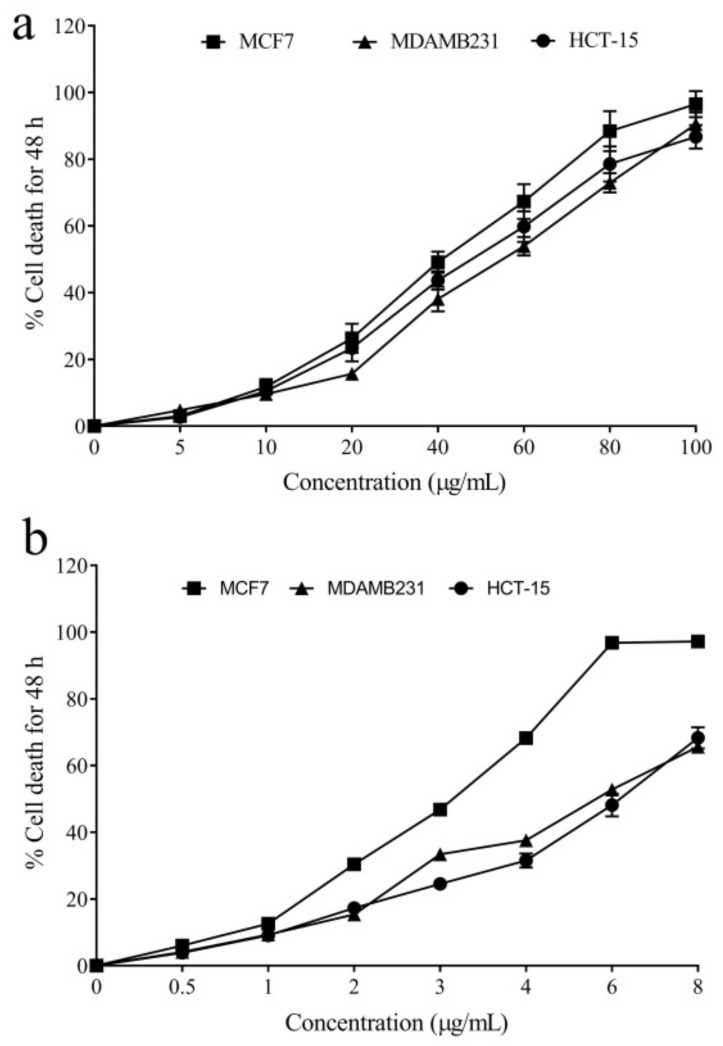
The anticancer potentials of the leaf extract of *A. nilagirica* (**a**) and cyclophosphamide (**b**).

Additionally, the results showed the anticancer properties of *A. nilagirica* in human breast and colon cancer cells. The anticancer activity was analyzed in three cancer cell lines, including MCF-7, MDA-MB-231, and HCT-15. We observed dose-dependent cytotoxicity in these three cell lines (Figure 2). The IC_50_ values against the three cells were estimated to be 41.79 ± 1.07, 55.37 ± 2.11, and 49.57 ± 1.46 µg/mL, respectively. In comparison, the standard cyclophosphamide was more toxic to these cells, with respective IC_50_ values of 3.12 ± 0.13, 5.74 ± 0.20, and 6.04 ± 0.21 µg/mL. Previous studies have also shown different species of *Artemisia* in various cancer cells [47,48,49,50]. In addition, the green synthesized nanoparticles from different *Artemisia* species are also reported to exert antiproliferative effects on cancer cells mediated through apoptotic cell death [23,51,52]. A study by Sahu, Meena, Shukla, Chaturvedi, Kumar, Datta, and Arya [24] indicated that ethyl acetate and hexane fractions of *A. nilagirica* induced cell death in colon, lung, and breast cancer cells. In addition, the bioactive compounds, including quercetin, apigenin, and eugenol, have also been shown to have significant antiproliferative effects by modulating different signaling pathways [53,54].

### 2.6. Anti-Inflammatory Activity of A. nilagirica

The *Artemisia nilagirica* extract was shown to inhibit the production of nitric oxide radicals in vitro. Further, the pretreatment of the extract also inhibited cytokine production and inflammatory insults in lipopolysaccharide-stimulated macrophages. The LPS is a microbial component that is known to stimulate inflammatory insults [55,56]. The Artemisia *nilagirica* leaf ethanol extract (AN) was found to inhibit the lipopolysaccharide-induced activation of macrophages and the subsequent cytokine release. The level of IL-1β was found to be significantly increased after LPS stimulation in macrophages; however, the pretreatment with AN at different doses significantly brought down the IL-1β levels in the macrophages (Table 8). Likewise, the levels of IL-6 and TNF-α also showed a similar increase during LPS exposure, which were successfully brought down by the treatment with different concentrations of *A. nilagirica*. The level of nitric oxide was determined biochemically and was also significantly elevated in LPS control cells. Pretreatment with 2.5, 5.0, and 7.5 µg/mL of AN successfully brought down the levels to 40.7 ± 1.6 (*p* < 0.05), 32.2 ± 2.4 (*p* < 0.05), and 25.7 ± 2.1 (*p* < 0.01). In addition, it is noted that the high dose of the extract resulted in stronger anti-inflammatory molecules compared to quercetin, which is a well-known anti-inflammatory molecule [57,58]. The LPS is known to stimulate cytokine production in macrophages by upregulating the NF-κB translocation to the nuclear compartment [59,60]. It is, therefore, possible that the *A. nilagirica* extract may also influence the LPS-induced activation of intracellular NF-KB signaling.

Thus, the study concludes that the *Artemisia nilagirica* ethanol extract exhibits antioxidant and anti-inflammatory properties in vitro and cultured cells. Further, the extract is also capable of inhibiting the proliferation of various cancer cells. The inhibition of enzymes associated with type 2 diabetes mellitus is also indicative of its anti-diabetic properties. The biological properties of the plant are expected to be due to the bioactive compounds identified in the *A. nilagirica* extract.

## 3. Materials and Methods

### 3.1. Artemisia Nilagirica (C.B.Clarke) Pamp. Collection and Extraction Using 100% Ethanol

The *Artemisia nilagirica* plant samples were collected from the Wayanad District, Kerala (11.7917° N, 76.1716° E). The mature leaves were carefully cleaned of all kinds of dust via washing. These leaves were dried under shade for 2 weeks and powdered using a mixer grinder; the powder was extracted with 100% ethanol using the Soxhlet method. Briefly, 100 g of the powder was extracted with ethanol at 80 °C for 8 h and the extract was collected, filtered, and concentrated before storage.

### 3.2. Phytochemical Analysis of Artemesia nilagirica

The leaf powder of *A. nilagirica* was analyzed for the proximate composition according to the methods used by Shukla et al. [61]. The qualitative phytochemical screening was carried out for the detection of alkaloids, flavonoids, glycosides, sterols, and triterpenes by referring to standard protocols [62,63]. The LC-MS analysis (Shimadzu LC- 8045, Kyoto, Japan) was used for phytochemical screening [64]; briefly, the C18 column measuring 4.6  ×  150 mm and 5 μm in size was used for the study, with methanol (A) and water with 0.1% formic acid (B) as the mobile phase (gradient elution mode). The gradient was set as 95% solution A (0–5 min), 70% solution A (5 to 10 min), 65% solution A (10 to 20 min), 50% solution A (20 to 30 min), and 90% of solution B (until 50 min), with a flow rate of 1.0 mL/min.

The quantitative profiling was estimated in terms of the total polyphenols [65] and total flavonoids [66], and the concentrations of ferulic acid, luteolin, caffeic acid, quercetin, and apigenin were determined using an HPLC analysis according to the same LC-MS conditions mentioned above.

### 3.3. Analysis of the Antioxidant Activity of A. nilagirica Ethanol Extract

The antioxidant activities were determined as the scavenging potentials of different radicals, including diphenyl picryl hydrazyl (DPPH), ABTS [67], and hydrogen peroxide [68]; the reducing potential on ferric ions was also estimated using the procedures described in [69]. The nitric oxide radical removal rate was used as an indicator of the inflammatory process inhibition model [70]. The DPPH was dissolved in methanol (0.1 mM) and varying concentrations of the extract were mixed with it. The solution was incubated for 20 min in the dark at 30 °C and the change in absorbance was used to estimate the percentage inhibition. Likewise, the ABTS radical generated was mixed with different doses of the *A. nilagirica* extract and the % inhibition was calculated spectrophotometrically. The nitric oxide scavenging was determined using sodium nitroprusside (8 mM) as the radical source; the Griess reagent was used to estimate the nitrite remaining in the treated samples using spectrophotometry at 596 nm.

Ascorbic acid was used as a positive control and standard for the antioxidant assays. The percentage inhibition was determined using the formula

$$Percentage\;inhibition=\frac{Absorbance\;of\;Control-Absorbance\;of\;Sample}{Absorbance\;of\;Control}\times 100$$

### 3.4. Efficacy of A. nilagirica Ethanol Extract on Activities of Enzymes

The enzyme-inhibitory properties were analyzed against the selected enzymes involved in diabetes and secondary diabetic complications. The inhibitory effect on α-amylase [71], α-glucosidase [72], aldose reductase [73], and sorbitol dehydrogenase [46] was assessed according to the standard methods.

### 3.5. Effect of A. nilagirica Ethanol Extract on Cancer Cell Proliferation

The human breast cancer cell lines MCF7 and MDA-MB-231 and a colon cancer cell line (HCT-15) were collected from NCCS, Pune, India. These cells were maintained in complete MEM, Leibovitz’s L-15, and RPMI-1640 media. The cells were selected as they are widely used in the anticancer screening of phytochemicals.

The inhibitory potential of the extract on human cancer cell proliferation (MCF7, MDA-MB-231, and HCT-15) was assessed using the MTT assay [74]. The IC_50_ value was determined using probit analysis.

### 3.6. Effect of A. nilagirica Extract on Lipopolysaccharide-Induced Cytokine Production in Macrophages

The murine Raw 264.7 cells were allowed to attach (1 × 10^7^ cells/mL) in a 24-well plate in complete growth media. The RPMI-1640 media was used to dilute the different concentrations of *A. nilagirica* (AN) (2.5, 5.0, and 7.5 µg/mL). Next, the cells were exposed to 1 µg/mL lipopolysaccharide for another 24 h. The protein expression of cytokines such as interleukin-1β and interleukin-6 and the tumor necrosis factor-α release were determined using PeproTech ELISA kits (Rocky Hill, CT, USA), as per the commercially prescribed methods. The nitric oxide release was quantified using the Griess reaction method [64]. Quercetin was used as a standard anti-inflammatory compound in the study.

### 3.7. Presentation of the Data, Software Used, and Statistical Analysis

The accuracy of the results obtained was ensured by conducting three independent assignments, with each having four replicates. Microsoft Excel 2010 was used for data consolidation and verification. The processed data are presented as means ± standard deviations; the IC_50_ values were estimated using probit analysis (GraphPad Prism 7.0, San Diego, CA, USA).

## 4. Conclusions

*Artemisia nilagirica* is an ethnomedicinal plant in India. In our study, the ethanol extract of *A. nilagirica* leaves showed significant antiradical and reducing potentials, which are indicative of its antioxidant potential. The IC_50_ values were lower but comparable with those of the standard ascorbic acid. The extract also inhibited enzymes associated with diabetes mellitus, including alpha-amylase and α-glucosidase. Additionally, the extract treatment significantly reduced the proliferative potential of breast and colon cancer cells. In Raw 264.7 macrophages, the pretreatment with the extract inhibited the LPS-stimulated production of cytokines and proved itself to be anti-inflammatory. Most importantly, the higher dose of the extract caused significantly higher activity than the standard quercetin used. Hence, we conclude that the ethanol extract of *A. nilagirica* leaves has antioxidant, anti-inflammatory, and anticancer properties; further studies on animal models and with bioassay-guided purification are necessary to identify the bioactive components.

## Figures and Tables

**Table 1:** Physicochemical parameters of *A. nilagirica* leaf powder.

Physicochemical Parameters	Result
Moisture content (%)	87.4 ± 2.12
Carbohydrate (%)	55.80 ± 4.1
Protein (%)	3.90 ± 0.16
Crude fat (%)	2.12 ± 0.18
Ash content (%)	0.74 ± 0.04

**Table 2:** Phytochemical constituents in the ethanol extract of *A. nilagirica*.

Test	Reaction
Alkaloids	
Marqui’s test	++
Wagner’s test	++
Mayer’s test	+++
Hager’s test	+
Froehde’s test	++
Dragendorff test	++
**Glycosides**	
Legal’s test	+
Keller-Kiliani test	+
**Flavonoids**	
Alkaline reagent test	++
Lead acetate test	++
Shinoda’s test	+++
**Tannins**	
Ferric Chloride test	++
Gelatin test	++
**Phytosterols**	
Salkowski’s test	++
Liebermann-Burchard test	+++
**Saponins**	
Froth test	+
Foam test	+
**Carbohydrates**	
Fehling test	++
Molish test	++
Benedict’s test	++
**Phenols**	
Folin-Ciocalteu test	+++
**Resin**	
Acetone-water test	+
**Fixed oils and fats**	
Stain test	-
**Triterpenes**	
Liebermann-Burchardt’s test	+++

Note: +++ high level, ++ moderate level, and + low-level presence of the compound.

**Table 3:** LCMS profiling of *A. nilagirica* with the retention time (RT), molecular mass, and chemical formula.

Sl. No.	RT (mins)	Compound Name	Formula	Mass
1	2.53	Ferulic acid	C_10_H_10_O_4_	194.00
2	6.38	Eugenol	C_10_H_12_O_2_	164.08
3	8.18	Β-caryophyllene	C_21_H_20_O_11_	448.40
4	9.06	Luteolin	C_15_H_10_O_6_	286.00
5	10.71	caffeic acid	C_9_H_8_O_4_	180.16
6	11.29	Quercetin	C_15_H_10_O_7_	302.00
7	12.14	Myricetin	C_15_H_10_O_8_	318.00
8	12.89	Apigenin	C_15_H_10_O_5_	270.05
9	14.03	Luteolin 5-0-beta-d-glucopyranoside	C_21_H_20_O_11_	448.13
10	15.52	Kaempferol	C_15_H_10_O_6_	286.23
11	21.56	Carnosic acid	C_20_H_28_O_4_	332.19
12	25.09	Artemisinin	C_20_H_20_O_8_	388.11
13	29.36	2alpha, 3 beta-Dihydroxyolean-12en-28-oic acid	C_30_H_48_O_4_	472.35
14	30.45	Menthyl acetate	C_12_H_22_O_2_	198.16
15	33.61	Oleanolic acid	C_30_H_48_O_3_	456.36
16	44.12	Basilimoside	C_36_H_60_O_6_	588.47

**Table 4:** The total polyphenol and flavonoid contents of *A. nilagirica* ethanol extract.

Assay	mg Equivalent/g
Total phenolic content	89.51 ± 2.5
Total flavonoid content	14.35 ± 0.9

**Table 5:** The quantification of selected compounds in the extract via HPLC.

RT (mins)	Compound Name	Quantity (µg/g Extract)
2.50	Ferulic acid	18.51 ± 1.82
9.05	Luteolin	146.87 ± 5.29
10.70	caffeic acid	88.62 ± 1.30
11.30	Quercetin	240.39 ± 4.87
12.87	Apigenin	103.41 ± 3.35

**Table 6:** In vitro antioxidant activities of *A. nilagirica* extract (AN) expressed as IC_50_ values (µg/mL).

Antioxidant Activity	IC_50_ Value (µg/mL)	
	AN	Ascorbic Acid
DPPH scavenging	23.12 ± 1.28	9.64 ± 0.89
ABTS scavenging	27.44 ± 1.88	35.19 ± 1.47
H_2_O_2_ scavenging	12.92 ± 1.05	19.08 ± 1.65
FRAP value (EC_50_)	5.42 ± 0.19	3.22 ± 0.15
Nitric oxide scavenging	367.09 ± 12.05	68.10 ± 2.11

**Table 7:** In vitro enzyme-inhibitory properties of *A. nilagirica* expressed as IC_50_ values (µg/mL).

Enzyme	IC_50_ Value (µg/mL)
α-Amylase	38.42 ± 2.71
α-Glucosidase	55.31 ± 2.16
Aldose reductase	17.42 ± 0.87
Sorbitol dehydrogenase	29.57 ± 1.46

**Table 8:** Effect of the *Artemisia nilagirica* leaf ethanol extract (AN) against lipopolysaccharide-induced macrophage (Raw 264.7) activation, cytokine release (in pg/mg protein), and nitric oxide production (µM/mg protein).

Nature		Tumor Necrosis Factor α	Interleukin 6	Interleukin 1β	NO
Untreated		97.6 ± 2.8	76.4 ± 3.1	67.8 ± 2.8	7.4 ± 0.57
Negative Control (LPS alone)		420.8 ± 10.6	795.2 ± 11.7	628.9 ± 14.2	52.1 ± 2.0
Quercetin (4.5 µg/mL)		279.1 ± 11.3 **	414.2 ± 10.7 ***	334.8 ± 11.7 **	30.7 ± 1.2 *
*Artemisia nilagirica* extract	2.5 µg/mL	314.1 ± 14.5 *	698.0 ± 17.3 **	477.6 ± 11.8 **	40.7 ± 1.6 *
	5.0 µg/mL	265.7 ± 10.7 **	524.3 ± 15.6 **	389.5 ± 14.6 **	32.2 ± 2.4 *
	7.5 µg/mL	190.9 ± 14.8 ***	388.2 ± 15.8 ***	298.7 ± 15.2 ***	25.7 ± 2.1 **

*Artemisia nilagirica* leaf ethanol extract (AN), lipopolysaccharide (LPS), nitric oxide (NO). The significance is indicated as * (*p* < 0.05), ** (*p* < 0.01), *** (*p* < 0.001).

## Data Availability

Data is contained within the article.

## Sample Availability

Samples of the compounds are available from the corresponding author.
